# Supplementary material for: Age-Stratified Trends in Nutrition and Lifestyle Transitions in Korea: Findings from KNHANES 2013–2022
Source: Nutrients. 2025 Oct 19;17(20):3282. doi: 10.3390/nu17203282 (PMC12567039; doi:10.3390/nu17203282)
Supplement: Supplementary file 1 [file nutrients-17-03282-s001.zip › nutrients-3912047-supplementary.pdf]

Supplementary Material

Supplementary Table S1. STROBE Statement—Checklist of Items for Cross-Sectional Studies

Study Title: *Age-Stratified Trends in Nutrition and Lifestyle Transitions in Korea: Findings from KNHANES 2013–2022*

	Section/Topic	STROBE Recommendation	*Page/Line Number*	How Item Is Addressed in This Study
Title and abstract				
1	**(a) Title**	Indicate the study's design with a commonly used term in the title or the abstract	*Title, Line 2-3*	The title explicitly states "Age-Stratified Trends" indicating a cross-sectional trend analysis design using KNHANES 2013–2022 data.
	**(b) Abstract**	Provide in the abstract an informative and balanced summary of what was done and what was found	*Lines 10–31*	The abstract includes: (1) Background statement (lines 10–11), (2) Study objectives (lines 12–13), (3) Methods including data source, sample size, and analytical approach (lines 14–18), (4) Main results with statistical significance (lines 18–26), and (5) Conclusions (lines 26–31).
Introduction				
2	**Background/rationale**	Explain the scientific background and rationale for the investigation being reported	*Lines 36–76*	The Introduction describes: (1) Korea's rapid demographic aging and dietary Westernization (lines 36–46), (2) Public health implications for chronic diseases (lines 48–59), (3) International evidence from Japan, China, and Europe (lines 60–69), and (4) Knowledge gaps justifying this investigation (lines 70–76).
3	**Objectives**	State specific objectives, including any prespecified hypotheses	*Lines 77–81*	The study objectives are explicitly stated: to examine temporal trends in dietary intake, anthropometric measures, biochemical markers, and lifestyle behaviors among Korean adults from 2013 to 2022, stratified by age group.
Methods				
4	**Study design**	Present key elements of study design early in the paper	*Lines 84–105*	The Methods section begins with a clear statement that this is a cross-sectional trend analysis using nationally representative KNHANES data from 2013–2022. STROBE compliance is explicitly noted (lines 84–101).
5	**Setting**	Describe the setting, locations, and relevant dates, including periods of recruitment, exposure, follow-up, and data collection	*Lines 87–101*	The setting is described as the Korea National Health and Nutrition Examination Survey (KNHANES), a nationwide cross-sectional survey conducted annually by the Korea Disease Control and Prevention Agency. Data collection periods (2013–2022) and survey methodology are specified.
6	**Participants**	Give the eligibility criteria, and the sources and methods of selection of participants	*Lines 95–100*	Eligibility criteria are specified: non-institutionalized Korean civilians aged ≥18 years who completed health examinations and dietary surveys. The multi-stage clustered probability sampling design is described. The final analytical sample of N=61,688 is reported with exclusions noted.
7	**Variables**	Clearly define all outcomes, exposures, predictors, potential confounders, and effect modifiers. Give diagnostic criteria, if applicable	*Lines 107–155*	All variables are systematically defined across subsections:<br>• Dietary assessment (lines 118–132): 24-hour recall method, nutrient calculations<br>• Anthropometric measures (lines 112–115): Height, weight, BMI, waist circumference<br>• Vital signs (lines 111–112): Blood pressure measurement protocols<br>• Biochemical markers (lines 133–139): fasting glucose, hemoglobin A1c, total cholesterol, triglycerides<br>• Behavioral factors (lines 140–155): Breakfast consumption, eating-out frequency, sedentary time<br>• Demographic variables (lines 107–111): Age groups, sex, income, living arrangement
8	**Data sources/measurement**	For each variable of interest, give sources of data and details of methods of assessment (measurement). Describe comparability of assessment methods if there is more than one group	*Lines 83–155*	Data sources and measurement methods are detailed for each variable domain. Standardized KNHANES protocols are referenced. Dietary data were collected through face-to-face interviews by trained dietitians using validated 24-hour recall methods. Anthropometric and vital sign measurements were performed by trained examiners following standardized protocols. Biochemical analyses were conducted at a certified central laboratory. All methods remained consistent across survey years, ensuring comparability.
9	**Bias**	Describe any efforts to address potential sources of bias	*Lines 119–175*	Multiple strategies to minimize bias are described:<br>• Complex survey weighting to account for non-response and ensure national representativeness (lines 163–165)<br>• Standardized data collection protocols by trained personnel (lines 119–120)<br>• Central laboratory analysis for biochemical markers (lines 134–136)<br>• Bonferroni correction for multiple comparisons (line 173-175)<br>• Discussion of residual confounding, recall bias, and survival bias (Discussion, lines 441–457)
10	**Study size**	Explain how the study size was arrived at	*Lines 95–101*	The study included all eligible KNHANES participants from 2013–2022 meeting inclusion criteria (N=61,688). No additional sampling was performed beyond the established KNHANES sampling framework, which is designed to provide nationally representative estimates. Sample size calculations were not applicable as this was a secondary analysis of existing surveillance data.
11	**Quantitative variables**	Explain how quantitative variables were handled in the analyses. If applicable, describe which groupings were chosen and why	*Lines 95–181*	Quantitative variables were handled as follows:<br>• Age stratification: Three groups (18–39, 40–64, ≥65 years) based on life-stage considerations<br>• Temporal grouping: Four periods (2013–2015, 2016–2018, 2019–2021, 2022) to assess trends<br>• Nutrient intake: Energy-adjusted using nutrient density method (nutrient per 1,000 kcal or % of energy)<br>• Continuous outcomes: Analyzed using survey-weighted linear regression<br>• Binary outcomes: Analyzed using survey-weighted logistic regression
12	**Statistical methods**	(a) Describe all statistical methods, including those used to control for confounding<br>(b) Describe any methods used to examine subgroups and interactions<br>(c) Explain how missing data were addressed<br>(d) If applicable, describe analytical methods taking account of sampling strategy<br>(e) Describe any sensitivity analyses	*Lines 156–181*	(a) **Confounding control:** Complex survey weights incorporated sampling design, non-response adjustment, and post-stratification to population totals<br>(b) **Subgroup analyses:** Age-stratified models examined trends separately for young (18–39), middle-aged (40–64), and older (≥65) adults. Time period interactions were tested.<br>(c) **Missing data:** Complete-case analysis was employed.<br>(d) **Sampling strategy:** All analyses incorporated survey weights, strata, and cluster variables using R procedures to account for the complex sampling design.<br>(e) **Sensitivity analyses:** Bonferroni correction for multiple comparisons; between-period contrasts (2013–2015 vs. 2020–2022) to assess COVID-19 impact
Results				
13	**Participants**	(a) Report numbers of individuals at each stage of study<br>(b) Give reasons for non-participation at each stage<br>(c) Consider use of a flow diagram	*Lines 183–204, Table 1*	(a) **Participant flow:** Total eligible participants from KNHANES 2013–2022: N=61,488. Sample sizes by survey period: 2013–2015 (n=18,047), 2016–2018 (n=19,417), 2019–2021 (n=18,704), 2022 (n=5,320). Age-stratified distributions provided in Table 1.<br>(b) **Non-participation:** Exclusions due to incomplete dietary recalls, missing anthropometric data, or age <18 years were minimal. KNHANES employs rigorous quality control to minimize non-response.<br>(c) **Flow diagram:** Not included as KNHANES uses a pre-established sampling framework with minimal attrition. Participant counts by period and age group are clearly presented in Table 1.
14	**Descriptive data**	(a) Give characteristics of study participants<br>(b) Indicate number of participants with missing data for each variable of interest	*Lines 184–204, Table 1*	(a) **Participant characteristics:** Table 1 presents weighted means/percentages for all key variables by age group and time period, including demographics, anthropometrics, vital signs, socioeconomic factors, dietary intake, biochemical markers, and behavioral factors.<br>(b) **Missing data:** Missing data rates were <5% for all variables due to KNHANES quality assurance protocols. Complete-case analysis was used.
15	**Outcome data**	Report numbers of outcome events or summary measures over time	*Lines 183–289, Figures 1–2, Table 1*	Summary measures for all outcome variables are presented with weighted means and 95% confidence intervals by age group and time period. All estimates incorporate survey weights and include p-values for trend tests. Between-period changes (2013–2015 to 2020–2022) are shown in Figure 2.
16	**Main results**	(a) Give unadjusted estimates and, if applicable, confounder-adjusted estimates and their precision<br>(b) Report category boundaries when continuous variables were categorized<br>(c) If relevant, consider translating estimates of relative risk into absolute risk	*Lines 183–289, Figures 1–2 Table 1*	(a) **Adjusted estimates:** All estimates are survey-weighted, incorporating complex sampling design which inherently adjusts for demographic confounders through post-stratification. Weighted means and 95% confidence intervals are presented. P-values for linear trends reported using Wald F-tests.<br>(b) **Category boundaries:** Age groups: 18–39, 40–64, ≥65 years; Time periods: 2013–2015, 2016–2018, 2019–2021, 2022<br>(c) **Absolute changes:** Percent changes from baseline (2013–2015) to endpoint (2020–2022) presented in Figure 2.
17	**Other analyses**	Report other analyses done—eg analyses of subgroups and interactions, and sensitivity analyses	*Lines 209–289, Figure 1-2, Discussion*	**Age-stratified analyses:** All outcomes examined separately for three age groups, revealing differential trends by age.<br>**Between-period contrasts:** Formal comparison of 2013–2015 vs. 2020–2022 periods (Figure 2) to assess pandemic-era changes.<br>**Sensitivity analyses:** Bonferroni correction applied; COVID-19 period analyzed separately (Discussion, lines 408–415).<br>**Interaction testing:** Age × time interactions examined.
Discussion				
18	**Key results**	Summaries key results with reference to study objectives	*Lines 292–309*	Key findings are concisely summarized in section 4.1 (Summary of Key Findings) including: declining energy and carbohydrate intake, increasing protein and fat intake, age-specific patterns in anthropometric and biochemical changes, rising breakfast skipping, declining eating-out frequency, and increased sedentary time.
19	**Limitations**	Discuss limitations of the study, taking into account sources of potential bias or imprecision	*Lines 439–457*	Limitations comprehensively discussed: cross-sectional design limitations, dietary recall bias, single-item sedentary behavior assessment, survival bias, residual confounding, and COVID-19 confounding. Direction and magnitude of potential biases addressed where feasible.
20	**Interpretation**	Give a cautious overall interpretation of results considering objectives, limitations, multiplicity of analyses, results from similar studies	*Lines 310–457*	Results interpreted cautiously within broader context: alignment with international trends (US, Europe, Japan, China), socioeconomic development implications, age-specific patterns, clinical significance, behavioral implications, COVID-19 impact, and multiple comparisons addressed through Bonferroni correction.
21	**Generalisability**	Discuss the generalisability (external validity) of the study results	*Lines 459–468, Conclusions*	Generalizability explicitly addressed: KNHANES uses complex probability sampling designed to be nationally representative; findings generalizable to Korean adult population but may not extend to institutionalized individuals or other countries; international comparisons suggest some trends may be generalizable to other post-industrial societies.
Other information				
22	**Funding**	Give the source of funding and the role of the funders for the present study	*Funding section*	This work was supported by the National Research Foundation of Korea (NRF-2022R1A2C2093323) grant funded by the Korea government (MSIT) (No. RS-2022-NR070518). The funders had no role in the study design, data collection and analysis, interpretation of results, decision to publish, or preparation of the manuscript.

Supplementary Note

This STROBE checklist was completed to ensure transparent reporting of this cross-sectional study. All 22 items of the STROBE Statement for cross-sectional studies have been addressed in the manuscript. Line numbers refer to the revised manuscript version submitted after peer review.

STROBE Statement Citation:
von Elm E, Altman DG, Egger M, Pocock SJ, Gøtzsche PC, Vandenbroucke JP; STROBE Initiative. The Strengthening the Reporting of Observational Studies in Epidemiology (STROBE) statement: guidelines for reporting observational studies. *J Clin Epidemiol.* 2008 Apr;61(4):344-9. doi: 10.1016/j.jclinepi.2007.11.008. PMID: 18313558.

Manuscript Correspondence: For questions regarding how specific STROBE items were addressed in this study, please contact the corresponding author.

END OF SUPPLEMENTARY TABLE S1

Generated: October 8, 2025
